# BL-DVDMS-mediated photodynamic therapy induces oxidative response associated with cellular senescence via p53/GADD45A signaling in lung cancer

**DOI:** 10.3389/fphar.2026.1935296

**Published:** 2026-10-28

**Authors:** Xing Li, Minghai Ma, Yibo Mei, Pan Zhang, Xiao Liang, Rou Huang, Yuanquan Zhang, Dalin He, Lei Wang

**Affiliations:** 1 Department of Thoracic Surgery, Tangdu Hospital, The Fourth Military Medical University, Xi’an, China; 2 Department of Urology, First Affiliated Hospital, Xi’an Jiaotong University, Xi’an, China

**Keywords:** cellular senescence, DNA damage, lung cancer, photodynamic therapy, sinoporphyrin sodium

## Abstract

Current chemotherapy options for lung cancer (LC) are limited by poor tumor selectivity, systemic toxicity, and acquired resistance. Photodynamic therapy (PDT) offers a promising minimally invasive modality as a potential conservative or complementary approach, yet its clinical adoption has been constrained by suboptimal photosensitizers and unclear mechanisms. This study investigated a novel PDT regimen combining a 450 nm blue laser with the photosensitizer sinoporphyrin sodium (DVDMS) to address these limitations in LC. Our findings demonstrate that BL-DVDMS treatment selectively inhibits lung cancer cell proliferation while sparing normal bronchial epithelial cells. Low-dose PDT induces a moderate oxidative stress response, leading to DNA damage and G2/M cell cycle arrest. Importantly, this treatment triggers a senescence-associated phenotype, evidenced by increased SA-β-Gal activity and elevated secretion of SASP factors (IL-6, IL-8), accompanied by altered p53/GADD45 A signaling. These effects are abrogated by ROS scavenging or p53 inhibition, indicating that the oxidative response plays a central role in the observed antitumor activity. In the subcutaneous xenograft model, this regimen significantly suppresses tumor growth with no detectable systemic toxicity. Collectively, this work identifies BL-DVDMS-mediated PDT as a mechanistically defined, senescence-associated therapeutic strategy. This study provides a potential complementary treatment option for inoperable LC patients, particularly for managing residual disease and enhancing existing therapies.

## 1 Introduction

Lung cancer remains the leading cause of cancer-related mortality worldwide, with approximately 2.4 million new cases and 1.8 million deaths recorded in 2022 (Bray et al., 2024). Histologically, non-small cell lung cancer (NSCLC) constitutes about 85% of all cases, distinct from small-cell lung cancer in both morphology and clinical behavior (Mo et al., 2024). Among these, central NSCLC originating in the trachea or main bronchi presents a unique therapeutic dilemma. Its proximity to vital mediastinal structures often renders tumors unresectable, while conventional radiotherapy and chemotherapy are frequently limited by toxicity and poor local control (Spicer et al., 2024). Although systemic advances in targeted therapy and immunotherapy have improved overall survival, they often fail to address endoluminal obstruction or prevent local recurrence in central airways (Lahiri et al., 2023; Fang et al., 2024). Given that these tumors arise within the bronchial tree, they are theoretically ideal candidates for local minimally invasive interventions (Hendriks et al., 2024). However, current options remain limited in their ability to secure local control, necessitating the development of novel adjuvant strategies to improve patient prognosis.

Photodynamic therapy (PDT) is an established minimally invasive modality for oncology (Rajan et al., 2024). Its efficacy hinges on light mediated activation of a photosensitizer (PS) in oxygenated tissue, which produces cytotoxic reactive oxygen species (ROS) that eliminate tumor cells by inducing apoptosis, disrupting tumor microvasculature, and regulating antitumor immunity (Aziz et al., 2023). Unlike systemic therapies, PDT offers superior spatial selectivity with minimal collateral damage, making it ideal for endoluminal malignancies accessible via endoscopy (Chen A et al., 2024). In central NSCLC, where surgical resection is often challenging, PDT under bronchoscope provides a critical strategy for local control by delivering light directly to tracheobronchial lesions (Hong and Chang, 2024). However, its clinical translation remains curbed by two persistent barriers: the shallow penetration depth of conventional excitation sources and the suboptimal pharmacokinetic profiles of existing photosensitizers (Kochergin et al., 2024). The core mechanism of PDT anti-tumor therapy is still unclear, which limits the optimization of mechanism-based treatment strategies (Cai et al., 2025). Resolving these critical limitations requires not only deeper-penetration light sources and potent photosensitizers but also thorough mechanistic exploration of the underlying signaling pathways, which is essential to fully realize the therapeutic potential of PDT against central airway malignancies.

Building on extensive research into laser-tissue interactions, our group developed a novel 450-nm BL surgical system. Preliminary investigations have demonstrated that BL not only exhibits favorable tissue penetration for effective tissue vaporization and dissection, but also serves as a potent excitation source for PDT (Xu et al., 2019; Wu et al., 2023; Li et al., 2022). Notably, this system has received regulatory approval for clinical application and research from the National Medical Products Administration of China (No. 20213010922), establishing a solid foundation for translational studies (Xu J et al., 2022; Jiang et al., 2023). Complementing this light source, sinoporphyrin sodium (DVDMS) emerges as an ideal photosensitizer candidate. Derived from Photofrin II, DVDMS is an ether-linked porphyrin dimer sodium salt characterized by superior chemical stability, high aqueous solubility, and rapid systemic clearance, which collectively enhance its safety profile compared to first-generation agents (Mai et al., 2020). Critically, the absorption spectrum of DVDMS exhibits a strong overlap with the 450-nm emission of the BL (Li et al., 2022). Therefore, the combination of this BL and DVDMS represents a promising PDT strategy.

Cellular senescence is an irreversible state of growth arrest triggered by diverse stressors, including oxidative stress, DNA damage, telomere attrition, oncogene activation, and mitochondrial dysfunction (Mchugh et al., 2025). Given its capacity to permanently suppress proliferation, inducing senescence has emerged as a safe and viable therapeutic strategy for cancer control (Colucci et al., 2025). Mechanistically, PDT exerts its anticancer effects largely through the generation of ROS, which impose severe oxidative stress on target cells (Wang Y et al., 2025). Crucially, cellular responses to PDT depend heavily on treatment dose. High light doses generally induce rapid apoptosis or necrosis, whereas low-level reactive oxygen species produced by mild, non-lethal PDT do not immediately kill cells outright and may instead trigger cellular senescence (Zheng et al., 2024; Yan et al., 2026). This suggests that modulating PDT dosimetry to favor senescence induction offers a feasible avenue, potentially enhancing treatment safety by sparing surrounding normal tissues from acute cytotoxic damage while effectively suppressing tumor proliferation.

In this study, we provide the first evidence that PDT mediated by the 450-nm BL–DVDMS combination induces cellular senescence in human LC cells via ROS-dependent mechanisms. Beyond characterizing this phenotypic shift, we further elucidate the underlying molecular pathways governing this process. Collectively, our findings will provide new insights for developing effective PDT strategies for LC.

## 2 Materials and methods

### 2.1 Reagents and irradiation

Sinoporphyrin sodium (DVDMS) was supplied by Jiangxi Qinglong Hi-tech Co., Ltd (Yichun, China). Stock solutions (1.25 mg/mL) were prepared in sterile physiological saline, aliquoted, and stored at −20 °C protected from light. Commercial assay kits were sourced as follows: Cell Counting Kit-8 (CCK-8) from TargetMol (MA, United States); 2′,7′-dichlorodihydrofluorescein diacetate (DCFH-DA) and N-acetylcysteine (NAC) from KeyGen BioTECH (Nanjing, China) and Selleck (Shanghai, China), respectively; Senescence β-galactosidase Staining Kit and Comet Assay Kit from Beyotime (Shanghai, China); ELISA kits from Elabscience (Wuhan, China); and Cell Cycle Analysis Kit from MedChemExpress (NJ, United States). All other chemicals were of analytical grade.

### 2.2 Light source

Photodynamic activation was performed using a 450-nm semiconductor laser system (Blueray Medical Ltd., Xi’an, China).

### 2.3 Cell culture

The human lung squamous cell carcinoma line (NCI-H838) and normal bronchial epithelial line (BEAS-2B) were obtained from the Cell Bank of Type Culture Collection, Chinese Academy of Sciences (Shanghai, China). The human lung adenocarcinoma line (A549) was provided by the Key Laboratory for Tumor Precision Medicine of Shaanxi Province (Xi’an, China).

Cells were maintained in respective specific media supplemented with 10% fetal bovine serum (Biological Industries, Israel) and 1% penicillin-streptomycin (New Cell and Molecular Biotech, Suzhou, China): NCI-H838 cells in 1,640 Medium (Procell, Wuhan, China), A549 cells in Ham’s F-12 K medium (Procell, Wuhan, China), and BEAS-2B cells in Eagle’s Minimum Essential Medium (Sigma-Aldrich, WI, United States). All cultures were incubated at 37 °C in a humidified atmosphere containing 5% CO_2_. For long-term storage, cells were cryopreserved in serum-free freezing medium (New Cell and Molecular Biotech, Suzhou, China) and stored in liquid nitrogen.

### 2.4 Cell viability assay

Cell viability was assessed using the CCK-8 assay and Colony formation assay. Briefly, A549, NCI-H838, and BEAS-2B cells were seeded into 96-well plates at a density of 5 × 10^3^ cells/well and allowed to adhere overnight. Subsequently, cells were treated with varying concentrations of DVDMS (0, 0.1, 0.2, 0.4, and 0.8 μM) and incubated for 4 h in the dark. Following drug uptake, cells were irradiated with blue laser (BL) at a power density of 10 mW/cm^2^ for 600 s, delivering a total energy dose of 6 J/cm^2^. After an additional 24 h post-PDT incubation, 10 μL of CCK-8 solution was added to each well. Absorbance at 450 nm was recorded after a 2 h reaction period using a microplate reader.

For colony formation assay, cells were seeded at 500 cells per well in 6-well plates and allowed to attach overnight. Following PDT treatment, cells were cultured for 10–14 days. Colonies were fixed with methanol and stained with 0.5% crystal violet. Colonies containing ≥50 cells were counted and analyzed.

### 2.5 Measurement of ROS levels

To evaluate intracellular ROS generation, cells were preloaded with 10 μM DCFH-DA in serum-free medium for 1 h in the dark. Following probe loading, cells were washed with PBS to remove excess dye and immediately irradiated with BL. Fluorescence signals indicative of ROS levels was captured immediately post-irradiation using fluorescence microscopy and quantified via flow cytometry. To verify the role of ROS in PDT-induced cytotoxicity, an inhibition assay was performed using NAC, a potent ROS scavenger. Cells were pre-treated with 5 mM NAC for 1 h prior to DVDMS incubation and subsequent BL irradiation. NAC was removed by replacing with fresh medium before DVDMS incubation and irradiation. Post-PDT, both intracellular ROS levels and cell viability were assessed to determine the protective effect of NAC.

### 2.6 Comet assay

DNA damage was evaluated using the alkaline comet assay. Briefly, 1 × 10^4^ cells were mixed with 1% low-melting-point agarose and layered onto comet slides. Cells were lysed in lysis buffer (2.5 M NaCl, 100 mM EDTA, 10 mM Tris, 1% Triton X-100, pH 10) for 1 h at 4 °C, followed by alkaline electrophoresis (300 mM NaOH, 1 mM EDTA, pH > 13, 25 V, 30 min). Slides were neutralized, stained with GelRed, and analyzed using software. Olive tail moment was calculated for at least 50 cells per group.

### 2.7 Transcriptome sequencing

Total RNA was extracted from BL-PDT-treated A549 cells using TRIzol reagent. Quality control confirmed high integrity (RIN ≥7.0) prior to cDNA library construction. Libraries were sequenced on an Illumina platform (Novogene, Tianjin, China). Three independent biological replicates per group were analyzed to ensure statistical robustness. For RNA-seq analysis, differentially expressed genes were identified using DESeq2 with criteria of FDR-adjusted p-value <0.05 and |log_2_ fold change| ≥ 1. A p-value <0.05 was considered statistically significant.

### 2.8 Senescence-associated-β-galactosidase (SA-β-gal) staining

Cellular senescence was evaluated using the SA-β-Gal Staining Kit. Briefly, post-PDT cells were fixed with 4% paraformaldehyde for 15 min at room temperature, washed with PBS, and subsequently incubated with the SA-β-Gal staining solution (pH 6.0) overnight at 37 °C in a CO_2_-free environment. Senescence-associated blue-green precipitates were visualized using an inverted light microscope. For quantitative analysis, images were captured from at least three random fields per well, and the percentage of SA-β-Gal-positive cells was calculated relative to the total cell count.

### 2.9 ELISA

Culture supernatants were collected 48 h post-PDT and centrifuged at 1,000 × g for 10 min at 4 °C to remove cellular debris. IL-6 and IL-8 levels were measured using human IL-6 and IL-8 ELISA kits (Proteintech) according to the manufacturer’s instructions. Briefly, 96-well plates pre-coated with capture antibodies were incubated with standards or samples for 2 h at room temperature. After extensive washing with wash buffer, biotinylated detection antibodies were added and incubated for 1 h, followed by washing and incubation with horseradish peroxidase (HRP)-conjugated streptavidin for 30 min. The plates were then washed again, and TMB substrate solution was added and incubated in the dark for 15 min. The reaction was terminated by adding stop solution, and absorbance was read at 450 nm using a microplate reader. Cytokine concentrations were calculated from standard curves.

### 2.10 Cell cycle analysis

Cell cycle distribution was assessed via propidium iodide (PI) staining following RNase A digestion. Briefly, PDT-treated cells were harvested, washed with cold PBS, and fixed in 70% ice-cold ethanol at 4 °C overnight. Prior to staining, fixed cells were washed twice with PBS to remove residual ethanol and resuspended in a staining solution containing PI and RNase A (prepared according to the manufacturer’s protocol). After incubation in the dark for 30 min at room temperature, the DNA content of at least 10,000 events per sample was analyzed using a flow cytometer. Cell cycle phases were quantified using FlowJo software.

### 2.11 Western blot analysis

Twenty-four hours post BL-PDT, cells were harvested and lysed in radioimmunoprecipitation assay buffer supplemented with 1% phenylmethylsulfonyl fluoride on ice for 30 min. Protein concentrations were determined using a Bicinchoninic Acid assay kit. Equal amounts of protein (20 μg per lane) were denatured by boiling at 100 °C for 10 min, separated via SDS-PAGE, and electro-transferred onto polyvinylidene difluoride membranes. Non-specific binding was blocked with 5% non-fat milk in TBST for 1 h at room temperature. Membranes were subsequently incubated with specific primary antibodies overnight at 4 °C, followed by three washes with TBST and incubation with HRP-conjugated secondary antibodies for 1 h at room temperature. The following primary antibodies were used: anti-γ-H2AX (1:1,000, Abcam), anti-p53 (1:1,000, Abcam), anti-GADD45 A (1:1,000, abcam), anti-Cyclin B1 (1:1,000, Abcam), anti-CDK1 (1:1,000, Abcam), and anti-β-actin (1:5,000, Abcam). HRP-conjugated secondary antibodies were used at 1:5,000 dilution. Blots were developed using ECL substrate and imaged with a chemiluminescence imaging system. Protein bands were visualized using an Enhanced Chemiluminescence substrate and quantified using ImageJ software.

### 2.12 *In vivo* xenograft model and treatment protocol

Female BALB/c nude mice (4–5 weeks old, 18–20 g) were purchased from the Animal Experiment Center of Xi’an Jiaotong University (Xi’an, China) and housed under specific pathogen-free conditions with *ad libitum* access to food and water. Anesthesia was induced by placing mice in a chamber connected to a calibrated vaporizer with 4%–5% isoflurane in 100% oxygen at a flow rate of 2.0 L/min. Anesthesia was maintained via a nose cone with 1.5%–2.0% isoflurane in oxygen throughout the experimental procedures. Body temperature was maintained at 37 °C ± 0.5 °C using a heating pad throughout the procedure. At the end of the experiments, mice were anesthetized deeply with pentobarbital sodium (50 mg/kg, i. p.) and then euthanized by cervical dislocation. Euthanasia was performed by trained personnel, and death was confirmed by cessation of respiration and heartbeat.

To establish the xenograft model, A549 cells suspended in 0.1 mL PBS were subcutaneously injected into the right dorsal flank of each mouse. Tumor growth and body weight were monitored every other day. Tumor volume (V) was calculated using the formula: V = (length × width^2^)/2. Once tumors reached an average volume of ∼100 mm^3^, mice were randomly stratified into four groups (n = 6 per group): (1) control (0.9% saline, 0.1 mL), (2) BL (60 J/cm^2^), (3) DVDMS (2 mg/kg, 0.1 mL), and (4) PDT (DVDMS + BL). For the PDT and DVDMS groups, photosensitizer was administered via caudal vein injection, followed by laser irradiation at the tumor site 24 h post-injection. The control and Laser-only groups received equivalent volumes of saline. After 14 days of treatment, mice were humanely euthanized. Tumors were excised, photographed, and weighed. All animal procedures were performed in strict accordance with the Declaration of Helsinki principles, and approved by the Ethics Committee of Fourth Military Medical University (No. GKJ-Y-202403–081).

### 2.13 Immunohistochemistry

Tumor tissues were excised from xenograft mice, fixed in 4% paraformaldehyde, and embedded in paraffin. Sections (4 μm) were deparaffinized in xylene and rehydrated through graded ethanol series. Antigen retrieval was performed by heating sections in citrate buffer (pH 6.0) for 15 min. Endogenous peroxidase activity was blocked with 3% H_2_O_2_, and non-specific binding was blocked with 5% goat serum for 30 min at room temperature. Sections were incubated overnight at 4 °C with primary antibodies. The following primary antibodies were used: anti-γ-H2AX (1:200, Abcam), anti-p53 (1:200, Abcam), anti-GADD45 A (1:200, abcam). After washing with PBS, sections were incubated with HRP-conjugated secondary antibodies for 1 h at room temperature. Immunoreactivity was visualized using DAB substrate, and sections were counterstained with hematoxylin. Images were captured using a light microscope. Negative controls were performed by omitting the primary antibody.

### 2.14 Statistical analysis

Data are presented as mean ± standard deviation (SD) from at least three independent experiments (n ≥ 3). Statistical comparisons between two groups were performed using an unpaired two-tailed Student’s t-test, while differences among multiple groups were assessed by one-way analysis of variance (ANOVA). All statistical analyses and graphing were conducted using GraphPad Prism (version 10.2). Image quantification was performed using ImageJ. A p-value <0.05 was considered statistically significant. Significance levels are denoted as follows: *p < 0.05, * *p < 0.01, * * *p < 0.001.

## 3 Results

### 3.1 BL-mediated PDT inhibits the proliferation ability of tumor cells *in vitro*

We first evaluated the therapeutic efficacy of 450 nm BL-mediated PDT with the photosensitizer DVDMS in LC cells. As shown in Figure 1A, PDT induced a potent, dose-dependent reduction in LC cell viability at DVDMS concentrations from 0.1 to 0.8 μM. Notably, this effect was highly selective, with minimal impact on normal BEAS-2B cells under the same conditions. DVDMS alone, even at the highest concentration, showed negligible dark toxicity without irradiation. Although our previous work demonstrated that high-dose BL-PDT effectively induces apoptosis, concerns over photosensitivity-related adverse effects *in vivo* necessitate a more refined therapeutic strategy. Therefore, we employed sub-toxic doses of DVDMS (0.1 and 0.2 μM) to investigate the antitumor mechanisms of low-dose BL-PDT. Long-term inhibitory effects were confirmed by colony formation assays, which revealed a significant reduction in clonogenic survival post-treatment (Figures 1B,C).

**FIGURE 1:**
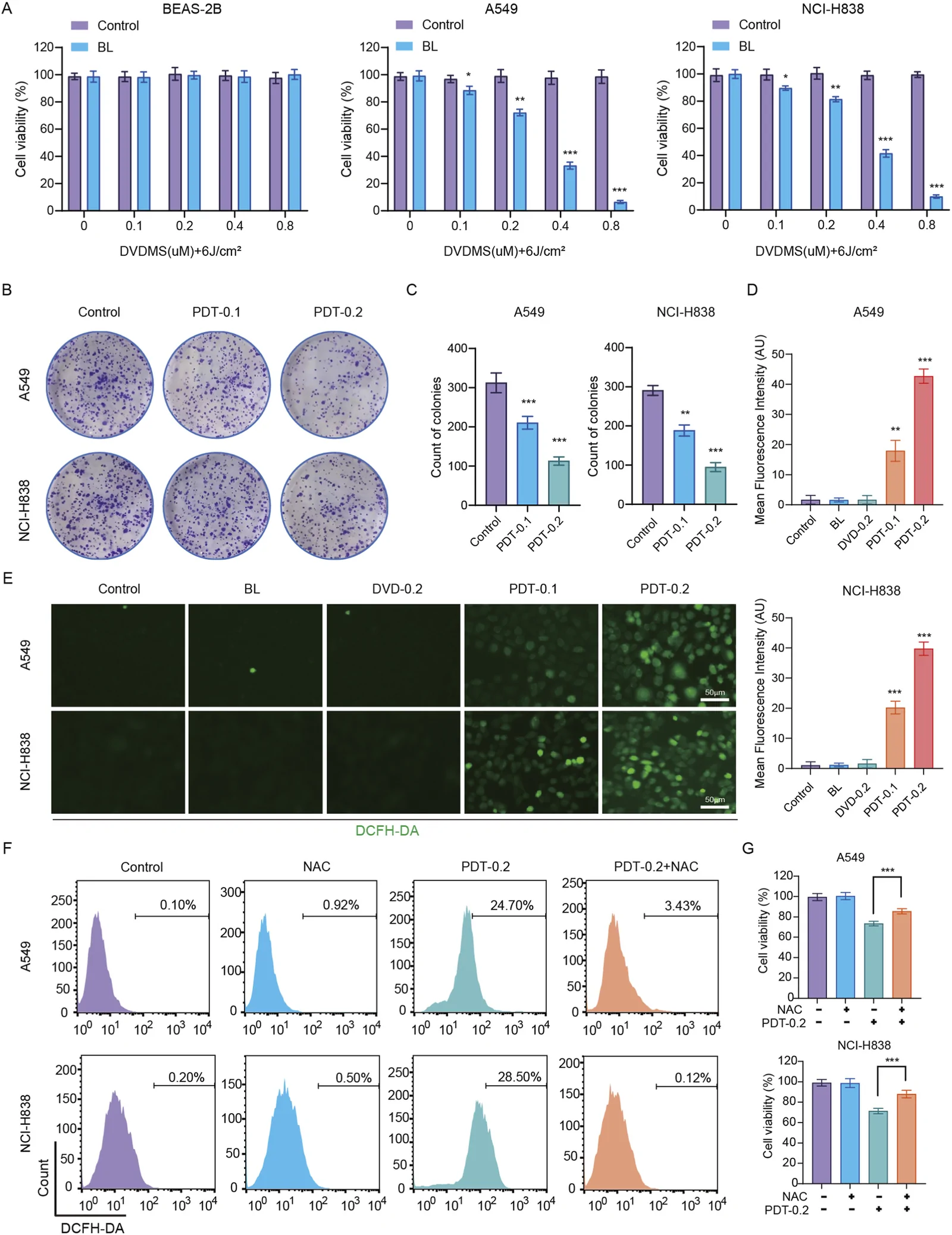
Anti-proliferative effects of BL-mediated PDT on LC cells. **(A)** BL-mediated photodynamic effect and cytotoxicity of DVDMS on cells. **(B,C)** Colony formation test after treating with BL-mediated PDT. **(D,E)** Representative fluorescence images and quantitative analysis of intracellular ROS detection. Scale bar = 50 μm. **(F)** Flow cytometry analysis of the ROS amount after adding the ROS inhibitor NAC. **(G)** Cell viability assay after adding the NAC. DVD-0.2: DVDMS concentration of 0.2, PDT-0.1/0.2: PDT at DVDMS concentration of 0.1/0.2 μM *p < 0.05, * *p < 0.01, * * *p < 0.001.

Since ROS are the principal mediators of PDT-induced cytotoxicity, we first quantified intracellular ROS accumulation following treatment. Fluorescence microscopy revealed a robust, DVDMS dose-dependent increase in ROS-specific fluorescence intensity in PDT-treated cells compared to controls (Figures 1D,E). This observation was corroborated by flow cytometry, which showed a marked elevation in ROS levels that was effectively abrogated by pretreatment with NAC (ROS scavenger) (Figure 1F). Crucially, NAC pretreatment significantly attenuated the PDT-induced suppression of cell viability. This rescue effect confirms that ROS generation is the indispensable driver of the antitumor efficacy observed in this BL-mediated PDT regimen (Figure 1G).

### 3.2 BL-mediated PDT induces γ-H2AX expression and DNA damage in LC cells

RNA-seq profiling of A549 cells revealed that BL-mediated PDT profoundly remodeled the transcriptome, yielding 2,595 differentially expressed genes (1,482 upregulated, 1,113 downregulated; Figure 2A). GO and GSEA analyses consistently enriched these DEGs in tumor-suppressive pathways, specifically cellular senescence, DNA damage response, and cell cycle arrest (Figures 2B,C). Heatmap analysis confirmed the significant upregulation of senescence and DNA damage markers (TP53, H2AFX, GADD45A, CDKN1A) alongside the downregulation of cell cycle drivers (CCNB1, CDK1, CDC25 C) (Figure 2D). Collectively, these data indicate that BL-mediated PDT exerts antitumor effects by triggering DNA damage-induced senescence and cell cycle arrest.

**FIGURE 2:**
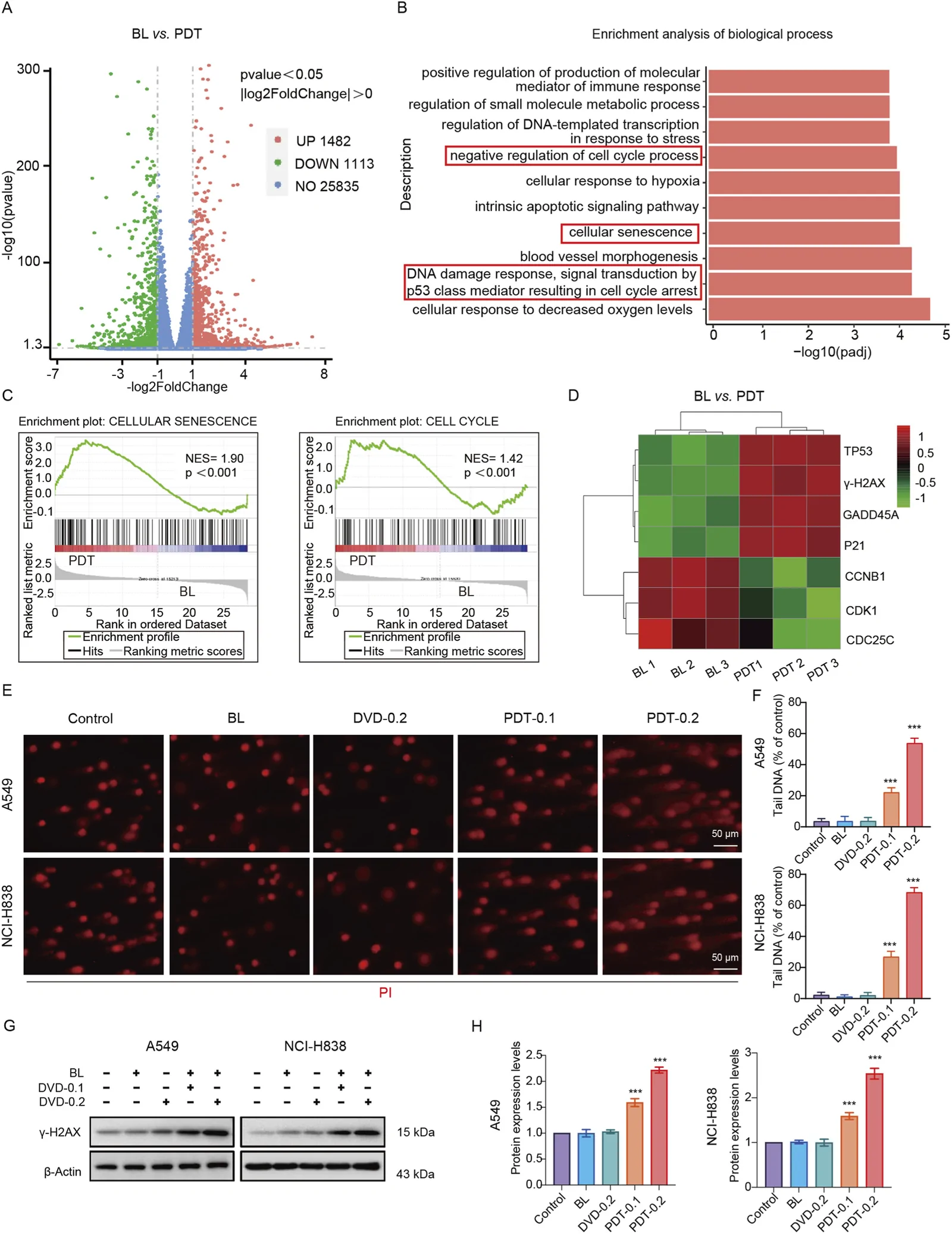
Bioinformatics analysis and DNA damage detection of BL-mediated PDT on LC cells. **(A)** Volcano plot showing differentially expressed genes (DEGs) in A549 cells between the BL group and PDT group. **(B,C)** GO and KEGG enrichment analyses of DEGs in A549 cells. **(D)** Heatmap plot of DEGs in A549 cells. **(E,F)** Representative fluorescence images and quantitative analysis of DNA damage. Scale bar = 50 μm. **(G,H)** Western blotting and quantitative analysis of DNA damage-related proteins expression levels. *p < 0.05, * *p < 0.01, * * *p < 0.001.

To determine whether DNA damage underpins the antiproliferative effects of BL-mediated PDT, we assessed genomic integrity using the alkaline comet assay. PDT-treated cells exhibited significant DNA strand breaks, characterized by pronounced comet tails that intensified in a DVDMS dose-dependent manner (Figures 2E,F). This physical evidence of genomic instability was corroborated at the molecular level by Western blotting, which showed a robust, dose-dependent upregulation of γ-H2AX, a sensitive marker of DNA double-strand breaks (Figures 2G,H). Collectively, these data confirm that BL-mediated PDT exerts its cytotoxicity by inducing severe DNA damage, thereby activating downstream growth inhibitory pathways.

### 3.3 BL-mediated PDT triggers G2/M cell cycle arrest and cellular senescence in LC cells

Western blot analysis revealed a significant decrease of G2/M phase arrest related proteins, Cyclin B1 and CDK1, in BL-mediated PDT-treated cells (Figures 3A,B), indicative of a checkpoint-mediated blockade preventing mitotic entry. This was corroborated by flow cytometry, which showed a marked dose-dependent expansion of the G2/M population (Figures 3C,D), confirming robust cell cycle arrest. Concurrently, PDT treatment induced a senescent phenotype, evidenced by a sharp increase in SA-β-Gal activity (Figures 3E,F). Furthermore, ELISA quantification revealed a significant elevation of the senescence-associated secretory phenotype (SASP) factors, IL-6 and IL-8, in the culture supernatants (Figures 3G,H). Collectively, these results demonstrate that BL-mediated PDT suppresses tumor proliferation by enforcing G2/M arrest and driving irreversible cellular senescence.

**FIGURE 3:**
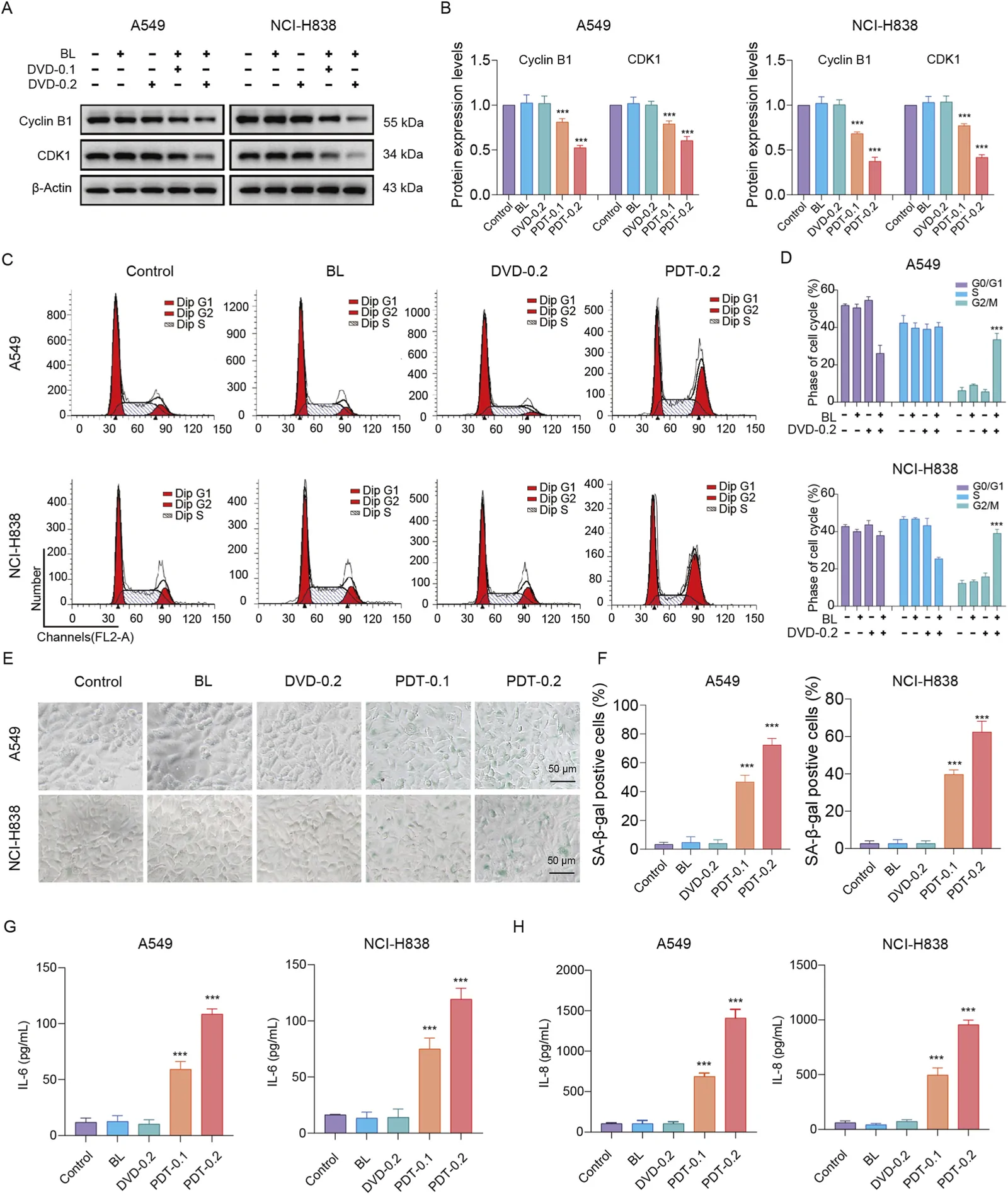
Cell cycle and senescence detection of BL-mediated PDT on LC cells. **(A,B)** Western blotting and quantitative analysis of cell cycle arrest-related protein expression levels. **(C,D)** Flow cytometry assay and analysis of the cell cycle in LC cells. **(E,F)** Detection and quantitative analysis of cellular senescence in LC cells. Scale bar = 50 μm. **(G,H)** ELISA analysis of cellular senescence related indicators (IL-6 and IL-8) in cell culture supernatant. *p < 0.05, * *p < 0.01, * * *p < 0.001.

### 3.4 ROS play a critical role in BL-mediated PDT-mediated inhibition of LC cells

To validate the causal role of ROS, NAC pretreatment was applied, which significantly attenuated PDT-induced genomic instability by reducing DNA damage (Figures 4A,B) and reversing γ-H2AX elevation (Figures 4C,D). Consequently, NAC abrogated the SASP, markedly reducing IL-6 and IL-8 secretion (Supplementary Figures S1A,B). Furthermore, ROS scavenging alleviated G2/M cell cycle arrest, restoring the homeostatic levels of key regulatory proteins, Cyclin B1 and CDK1 (Figures 4E–H). Collectively, these rescue experiments confirm that ROS generation is the upstream trigger that drives DNA damage, subsequently enforcing cell cycle arrest and senescence-associated phenotype in LC cells.

**FIGURE 4:**
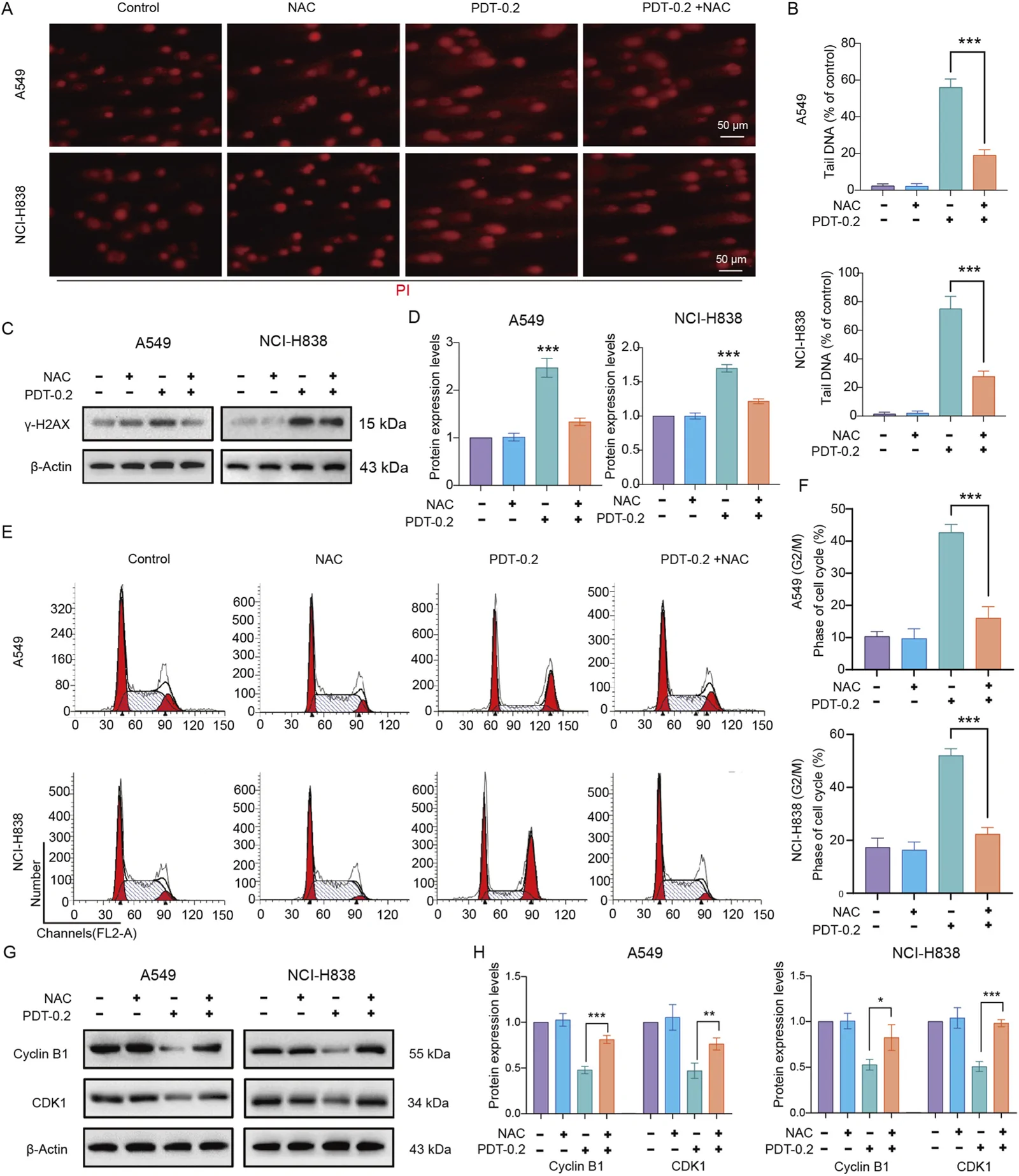
Effects of intracellular ROS suppression on DNA damage and cell cycle arrest induced by BL-mediated PDT in LC cells. **(A,B)** Representative fluorescence images and quantitative analysis of DNA damage after adding the NAC. Scale bar = 50 μm. **(C,D)** Western blotting and quantitative analysis of the expression levels of DNA damage-related proteins after adding the NAC. **(E,F)** Flow cytometry assay and analysis of the cell cycle after adding the NAC. **(G,H)** Western blotting and quantitative analysis of the expression levels of cell cycle arrest-related proteins after adding the NAC. *p < 0.05, * *p < 0.01, * * *p < 0.001.

### 3.5 BL-mediated PDT induces cellular senescence associated with p53/GADD45 A signaling axis

KEGG enrichment analysis identified the p53/GADD45 A signaling pathway as a potential mediator associated with BL-mediated PDT-induced senescence-associated phenotypes (Figures 5A,B). Consistent with this prediction, Western blotting revealed that BL-mediated PDT triggered a robust upregulation of p53 and GADD45A, an effect not observed with BL or DVDMS monotherapy (Figures 5C,D). To further explore this association, we employed the p53-specific inhibitor Pifithrin-α (PFT-α). PFT-α pretreatment attenuated the PDT-induced activation of the p53/GADD45 A axis (Figure 5E) and consequently reduced key senescence-associated phenotypes, including SA-β-Gal activity and the secretion of SASP factors (IL-6, IL-8) (Figures 5F–H; Supplementary Figure S1C). Furthermore, PFT-α reversed the expression profiles of cell cycle arrest-related proteins (Figure 5I). Collectively, these data demonstrate that BL-mediated PDT induces senescence-associated phenotypes in LC cells in a manner associated with p53/GADD45 A signaling.

**FIGURE 5:**
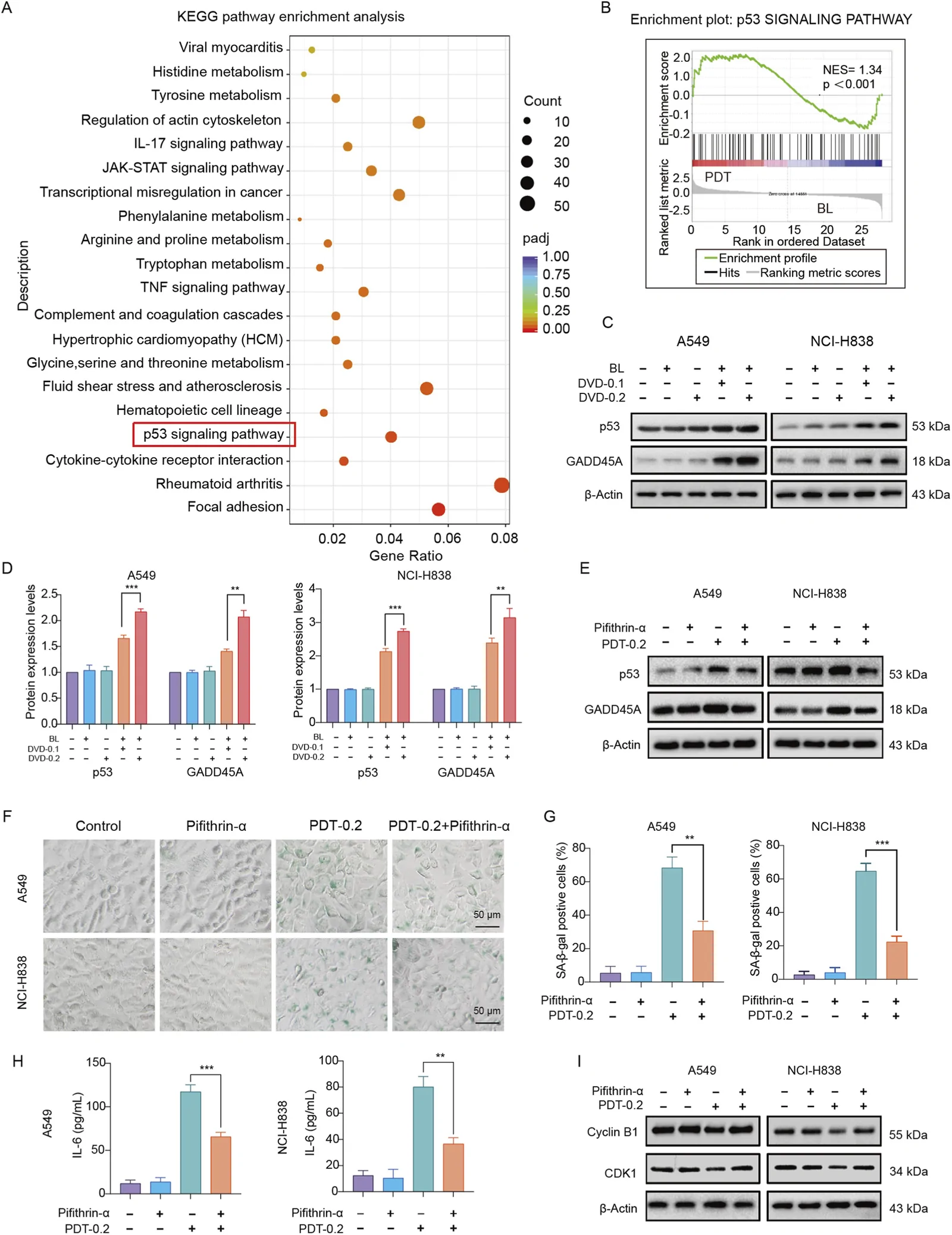
Activation of the p53/GADD45 A signaling pathway by BL-mediated PDT. **(A,B)** KEGG pathway enrichment analysis of differentially expressed transcripts in A549 cells. **(C,D)** Western blotting and quantitative analysis of pathway-related proteins expression levels. **(E)** Western blotting analysis of the expression levels of pathway-related proteins after adding the p53 inhibitor pifithrin-α. **(F,G)** Detection and quantitative analysis of cellular senescence after adding the pifithrin-α. Scale bar = 50 μm. **(H)** ELISA analysis of cellular senescence related indicators (IL-6) in cell culture supernatant after adding the pifithrin-α. **(I)** Western blotting and quantitative analysis of the expression levels of cell cycle arrest-related proteins after adding the pifithrin-α. *p < 0.05, * *p < 0.01, * * *p < 0.001.

### 3.6 BL-mediated PDT suppresses lung cancer *in vivo* via p53-dependent DNA damage

The *in vivo* therapeutic efficacy of BL-mediated PDT was evaluated in a subcutaneous A549 xenograft model using BALB/c nude mice. Following intravenous administration of DVDMS on day 14 post-implantation, tumors received local irradiation 24 h later (Figure 6A). BL-mediated PDT elicited a robust antitumor response, resulting in significant suppression of tumor progression compared to controls (Figure 6B). Quantitative metrics confirmed a marked reduction in both final tumor volume and weight (Figures 6C,D). Notably, this potent efficacy was achieved without compromising systemic safety, as evidenced by stable body weights across all groups (Supplementary Figure S2A) and the absence of histopathological abnormalities in major vital organs (heart, liver, spleen, lungs, kidneys) (Supplementary Figure S2B).

**FIGURE 6:**
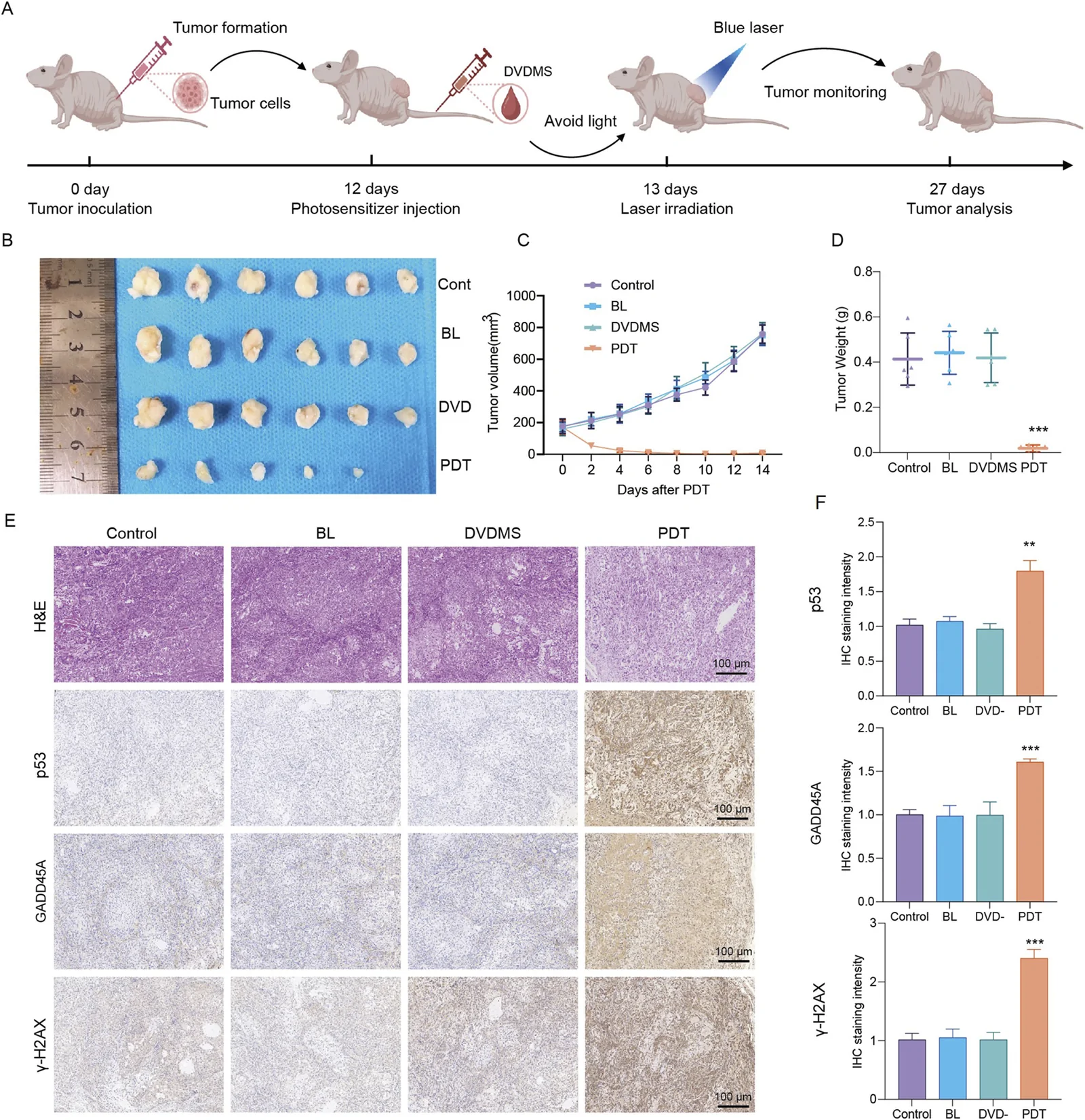
Anti-cancer effect of BL-mediated PDT *in vivo*. **(A)** Schematic of the time schedule for tumor implantation, DVDMS injection, laser irradiation, and monitoring of tumor growth. **(B)** Representative photos of tumor-bearing mice after different treatments. **(C)** Changes in tumor volume of mice during different treatments. **(D)** Changes in tumor weight after different treatments. **(E)** H&E staining and IHC analysis of tumors from the mice after different treatments. Scale bar = 100 μm. **(F)** Quantitative analysis of Figure E. *p < 0.05, * *p < 0.01, * * *p < 0.001.

Mechanistically, immunohistochemical analysis of excised tumors revealed that BL-mediated PDT was associated with activation of the p53/GADD45 A axis, characterized by marked upregulation of p53 and its downstream effector GADD45A, alongside elevated levels of the DNA damage marker γ-H2AX (Figures 6E,F). Collectively, these findings demonstrate that BL-mediated PDT effectively inhibits tumor growth in a subcutaneous xenograft model, with concomitant activation of p53/GADD45 A-mediated DNA damage responses and a favorable safety profile.

## 4 Discussion

Despite considerable advances in conventional LC therapies, the disease remains the leading cause of cancer-related mortality worldwide (Guo et al., 2024). There is therefore an urgent need for more effective and safer treatments that exert strong antitumor activity while minimizing damage to healthy tissue. Although PDT is a promising clinical approach, its clinical translation has been hindered by non-ideal photosensitizers, inefficient light delivery, and limited understanding of the mechanisms governing tumor selectivity (Cai et al., 2025). To address these issues, we developed a targeted PDT approach using a 450 nm BL combined with the photosensitizer DVDMS. Lung cancer cell proliferation *in vitro* and tumor growth *in vivo* were markedly suppressed by this combination, which also exhibited favorable safety profiles. Unlike conventional cytotoxic therapies, low-dose BL-PDT was found to exert antitumor effects primarily through irreversible cellular senescence rather than acute cell death, rendering it particularly suitable for high-risk central LC. Mechanistically, this response was driven by DNA damage and G2/M cell cycle arrest, mediated via the p53/GADD45 A signaling axis (Figure 7). By promoting stable growth arrest, this PDT regimen inhibited tumor progression and preserved normal tissue integrity, with no obvious systemic or organ toxicity detected *in vivo* animal models. Collectively, these results confirm the antitumor activity of BL-mediated PDT, clarify its molecular mechanism, and provide a rational basis for further optimization and clinical development as a senescence-targeted strategy against NSCLC.

**FIGURE 7:**
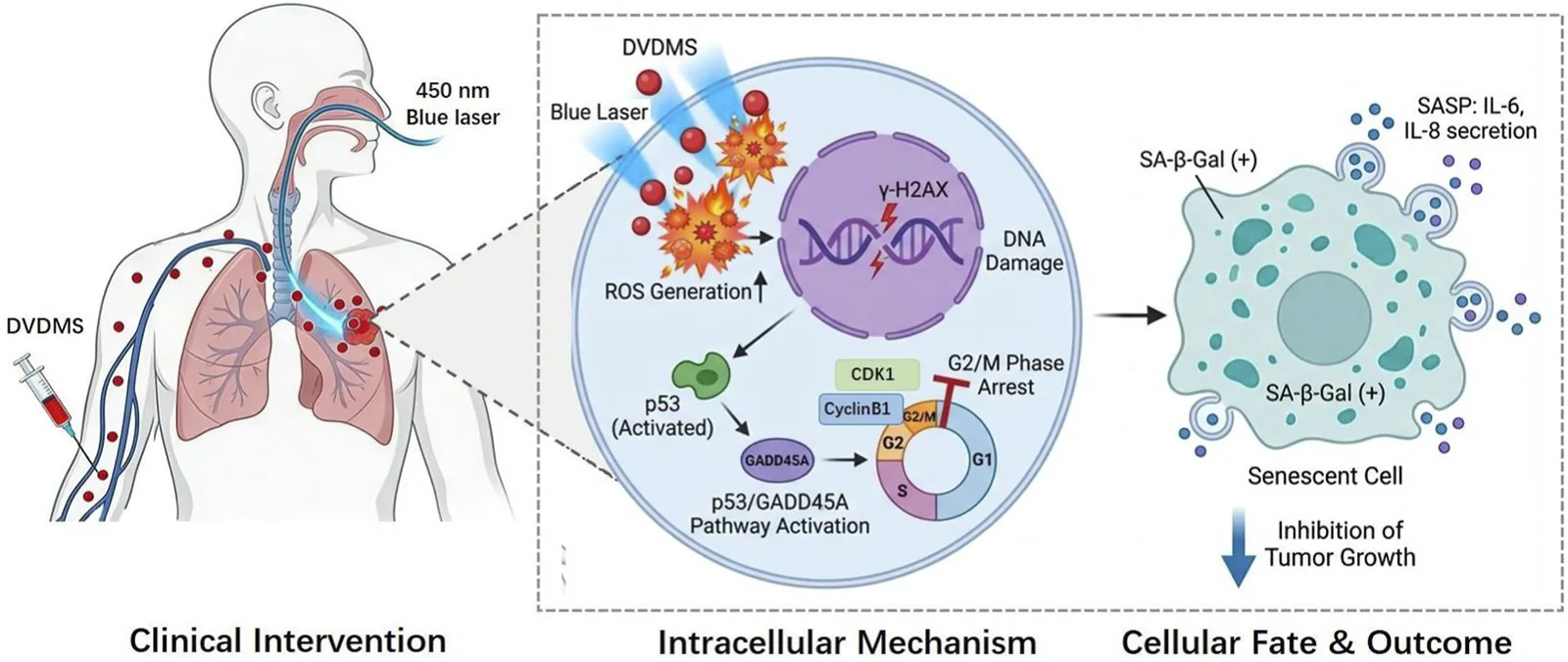
PDT mediated by sinoporphyrin sodium induces cellular senescence through ROS/p53/GADD45 A pathway in lung cancer.

Cellular senescence, a permanent state of cell cycle arrest triggered by DNA damage or oxidative stress, acts as a potent barrier to tumorigenesis by limiting proliferative capacity (Lu et al., 2024). This mechanism underpins the efficacy of many anticancer therapies, where therapy-induced senescence serves as a critical alternative to apoptosis for tumor suppression (Wang Z et al., 2025; Chen M et al., 2024). In PDT, the extent of cellular damage often determines the biological outcome. Sachsenmaier et al. observed that varying levels of PDT exposure could lead bone metastasis cells to undergo either apoptosis or senescence (Sachsenmaier et al., 2021). Consistent with this dose-dependent response, our data show that low-dose BL-PDT inhibits lung cancer progression specifically by inducing irreversible senescence via DNA damage and G2/M arrest. On the other hand, since central lung cancer is adjacent to vital anatomical structures including the trachea, large blood vessels and esophagus, acute necrosis or apoptosis induced by high-dose PDT may result in severe collateral damage to these critical tissues. Therefore, precise modulation of PDT dosage enables a shift in tumor-killing mechanisms from acute cytotoxicity toward sustained growth arrest, thereby improving both therapeutic efficacy and safety in treating central NSCLC.

DNA damage is a primary mechanism of anticancer therapies, including PDT (Sun et al., 2025). While severe damage triggers cell death, sub-lethal lesions often induce cell cycle arrest and senescence, a response observed with chemotherapy and radiotherapy (Gu et al., 2025; Xing and Stea, 2024). For instance, Bojko et al. demonstrated that agents like doxorubicin and oxaliplatin drive senescence in lung and other cancers via DNA damage (Bojko et al., 2019). To determine if low-dose BL-PDT operates through a similar pathway, we assessed DNA integrity in NSCLC models. Comet assays revealed significant tail fluorescence in PDT-treated cells compared to controls, indicating DNA strand breaks. Concurrently, immunoblotting showed markedly elevated γ-H2AX levels in both cells and tissues, confirming extensive double-strand breaks and disruption of DNA superhelical structure. These data directly link the senescent phenotypes observed in our study to DNA damage, supporting the conclusion that low-dose BL-PDT inhibits lung cancer growth by triggering a DNA damage-induced senescence program.

The p53 signaling pathway governs cell senescence by activating DNA repair mechanisms or triggering apoptosis upon irreparable damage, thereby maintaining genomic stability (Boon et al., 2024). Central to this process is p53-mediated transcriptional activation at G1 and G2/M checkpoints, which halts the cell cycle and initiates senescence (Colucci et al., 2025). GADD45A, a critical p53 downstream effector induced by genotoxic and oxidative stress, cooperates with p53 to enforce DNA repair, cell cycle arrest, and senescence (Palomer et al., 2024; Liu et al., 2025). Previous studies have demonstrated that GADD45 A silencing promotes proliferation while suppressing senescence in cutaneous squamous cell carcinoma, and its p53-dependent expression is essential for stress-induced growth arrest and apoptosis (Shrivastava and Ratnacaram, 2025; Liu et al., 2025). In our study, low-dose BL-PDT significantly upregulated both p53 and GADD45 A in NSCLC cells, coinciding with senescent phenotypes. Crucially, pharmacological inhibition of p53 abolished GADD45 A induction, reversed cell cycle arrest, restored proliferative capacity, and markedly reduced senescence rates. These findings confirm that BL-PDT induces senescence in NSCLC specifically through the activation of the p53/GADD45 A axis.

PDT primarily exerts antitumor effects through ROS generation, which triggers cell death, inhibits neovascularization, or stimulates immunity (Teng et al., 2025). While our previous work established that high-dose BL-PDT induces autophagy, apoptosis, and ferroptosis via massive ROS production, emerging evidence suggests sub-lethal ROS levels can drive senescence (Chen et al., 2023a; Chen et al., 2023b). Zu et al. demonstrated that low-dose ROS from emodin sensitizes breast cancer cells to chemotherapy by inducing DNA double-strand breaks and subsequent senescence (Zu et al., 2018). To elucidate this mechanism in BL-PDT, we measured ROS levels in A549 and NCI-H838 cells using DCFH-DA staining and flow cytometry. Low-dose BL-PDT elevated intracellular ROS, correlating with increased DNA double-strand breaks and senescent phenotypes. Crucially, pretreatment with the ROS scavenger NAC attenuated DNA damage, suppressed senescence, and blocked activation of the p53/GADD45 A pathway. These results establish a causal chain where low-dose BL-PDT generates moderate ROS levels that inflict DNA damage, thereby activating the p53/GADD45 A axis to enforce senescence and inhibit NSCLC growth.

Clinical evidence indicates that therapy-induced senescence following radiotherapy or chemotherapy correlates with improved patient prognosis (Prasanna et al., 2021). This benefit arises from a dose-dependent dichotomy where high-dose regimens trigger acute apoptosis while sub-lethal doses preferentially induce senescence (Xu X et al., 2022). Consequently, low-dose strategies offer a therapeutic advantage by minimizing systemic toxicity and preserving quality of life compared to conventional cytotoxic approaches (Wang et al., 2021; Dayes et al., 2023). Building on these findings, future work will investigate whether low-dose BL-mediated PDT can overcome drug resistance by enforcing senescence to establish a viable paradigm for combination therapy in refractory NSCLC.

However, we acknowledge several limitations of the *in vivo* model. The subcutaneous xenograft model does not recapitulate the anatomical complexity or optical limitations of central lung tumors, where light delivery to deep-seated lesions remains a major clinical challenge. The superficial tumor location and external illumination in our model bypass these challenges. Future studies using orthotopic lung cancer models or large animal models with endoscopic light delivery are warranted to evaluate the clinical feasibility of this approach for central lung cancer.

## 5 Conclusion

In summary, this study demonstrates that BL-DVDMS-mediated low-dose PDT effectively suppresses lung cancer progression by inducing ROS-dependent DNA damage and G2/M phase arrest, associated with activation of the p53/GADD45 A signaling axis and a senescence-associated phenotype. This shift from acute cytotoxicity toward sustained growth arrest achieves potent antitumor efficacy while sparing normal cells, as confirmed by both *in vitro* and *in vivo* experiments. Importantly, the regimen exhibits no systemic toxicity or organ damage in xenograft models, highlighting its favorable safety profile. These findings position BL-DVDMS-mediated PDT as a promising complementary treatment strategy for inoperable patients with unresectable lung cancer, particularly for managing residual disease and enhancing existing therapeutic regimens.

## Data Availability

The datasets presented in this article are not readily available because ongoing related follow-up studies. Requests to access the datasets should be directed to the corresponding authors.
