# Supplementary material for: BL-DVDMS-mediated photodynamic therapy induces oxidative response associated with cellular senescence via p53/GADD45A signaling in lung cancer
Source: Front Pharmacol. 2026 Oct 28;17:1935296. doi: 10.3389/fphar.2026.1935296 (PMC13752850; doi:10.3389/fphar.2026.1935296)
Supplement: Supplementary file 1 [file Supplementaryfile1.docx]

Supplementary Material

**Supplementary Figure 1**

Effects of intracellular ROS suppression on DNA damage and cell cycle arrest induced by BL-mediated PDT in LC cells. (A) ELISA analysis of cellular senescence related indicators (IL-6) in cell culture supernatant after adding the NAC. (B) ELISA analysis of cellular senescence related indicators (IL-8) in cell culture supernatant after adding the NAC.

**Supplementary Figure 2**

Activation of the p53/GADD45A signaling pathway by BL-mediated PDT. (A) ELISA analysis of cellular senescence related indicators (IL-8) in A549 cells culture supernatant after adding the pifithrin-α. (B) ELISA analysis of cellular senescence related indicators (IL-8) in NCI-H838 cells culture supernatant after adding the pifithrin-α.

v

**Supplementary Figure 3**

Anti-cancer effect of BL-mediated PDT in vivo. (A) Relative body weight changes of mice during different treatments. (B) H&E staining of vital organs of the mice after different treatments. Scale bar = 100 μm.
